# Reducing Agents Decrease the Oxidative Burst and Improve Clinical Outcomes in COPD Patients: A Randomised Controlled Trial on the Effects of Sulphurous Thermal Water Inhalation

**DOI:** 10.1155/2013/927835

**Published:** 2013-12-23

**Authors:** Marco Contoli, Giulia Gnesini, Giacomo Forini, Brunilda Marku, Alessia Pauletti, Anna Padovani, Paolo Casolari, Liliana Taurino, Andrea Ferraro, Milva Chicca, Adalberto Ciaccia, Alberto Papi, Silvano Pinamonti

**Affiliations:** ^1^Department of Medical Sciences, Research Centre on Asthma and COPD, University of Ferrara, Via Savonarola 9, 44121 Ferrara, Italy; ^2^Department of Life Science and Biotechnology, University of Ferrara, Via Savonarola 9, 44121 Ferrara, Italy

## Abstract

*Background*. Inhalation of thermal water with antioxidant properties is empirically used for COPD. *Aims*. To evaluate the effects of sulphurous thermal water (reducing agents) on airway oxidant stress and clinical outcomes in COPD. *Methods*. Forty moderate-to-severe COPD patients were randomly assigned to receive 12-day inhalation with sulphurous thermal water or isotonic saline. Patients were assessed for superoxide anion (O_2_^−^) production in the exhaled breath condensate and clinical outcomes at recruitment, the day after the conclusion of the 12-day inhalation treatment, and one month after the end of the inhalation treatment. *Results*. Inhalation of reducing agents resulted in a significant reduction of O_2_^−^ production in exhaled breath condensate of COPD patients at the end of the inhalatory treatment and at followup compared to baseline. A significant improvement in the COPD assessment test (CAT) questionnaire was shown one month after the end of the inhalatory treatment only in patients receiving sulphurous water. *Conclusion*. Thermal water inhalation produced an *in vivo* antioxidant effect and improvement in health status in COPD patients. Larger studies are required in order to evaluate whether inhalation of thermal water is able to modify relevant clinical outcomes of the disease (the study was registered at clinicaltrial.gov—identifier: NCT01664767).

## 1. Introduction

Chronic obstructive pulmonary disease (COPD) is a leading cause of morbidity and mortality worldwide and results in a substantial and increasing economic and social burden [1]. COPD is characterised by persistent airflow limitation that is usually progressive and that is associated with enhanced chronic inflammatory response of the airways and the lung to noxious particles or gases [1].

Oxidative stress is a key component of airway inflammation in COPD [2, 3]. In particular, the reactive metabolites of oxygen, such as superoxide anion (O_2_^−^) and the hydroxyl radical, are unstable molecules that can trigger significant oxidative processes at the cellular level. Such molecules can induce remodelling of the extracellular matrix and create a protease-antiprotease imbalance, and can alter cellular respiration, cell proliferation, surfactant production, the airway epithelial repair process and the immune response of the lung [2, 4, 5].

The regular use of antioxidant agents in COPD patients has been investigated in many long-term studies with conflicting results [6]. International guidelines report that the widespread of these agents cannot be recommended at present; that N-Acetyl-L-Cysteine (NAC) may have antioxidant effects; and that treatment with carbocisteine and NAC in COPD patients not receiving inhaled corticosteroids may reduce exacerbations [1].

The inhalation of thermal water with antioxidant properties is not considered to be a therapeutic option for COPD patients according to international guidelines [1]. However, the inhalation of thermal water has been used since the dawn of medicine to treat patients suffering from chronic disorders, including respiratory symptoms; such treatments are still used because they always have been. A “PubMed” search for the terms “COPD and thermal water inhalation” shows that there are only two placebo controlled studies focusing on the biological impact of thermal water inhalation on markers of airway inflammation in COPD patients [7, 8]. In several European countries, such as Italy, the costs of thermal water inhalation treatment for patients with COPD are reimbursed by the National Health System. In the era of evidence-based medicine and of economical restraint/cutting of budgets, cost/benefit analyses must be considered seriously.

The possible benefit of inhalation of thermal water for the treatment of chronic respiratory disorders has been mainly related to its supposed mucolytic, anti-inflammatory, and antioxidant properties [9–11]. Hydrogen sulphide (H_2_S) is a potent reducing agent that is present at high levels in sulphurous thermal waters and in lower concentrations in salt-bromide-iodine waters. Recently, we documented that treatment of bronchial epithelial cell lines with sulphurous thermal water before rhinovirus infection resulted in increased concentrations of the endogenous intracellular reducing agent GSH and in the inhibition of a rhinovirus-induced intracellular oxidant burst [12].

Based on these observations, we conducted a randomised controlled study to evaluate the effects of sulphurous thermal water (containing reducing agents) on airway oxidant stress, airway inflammation, and clinical outcomes in patients with COPD.

## 2. Material and Methods

### 2.1. Study Population

COPD patients were recruited at the outpatient clinic of the Respiratory Disease Section of the University of Ferrara, Italy.

The study population included 40 COPD patients, according to GOLD criteria [1]. We recruited patients aged 50–80 years old with a postbronchodilator FEV1/FVC ratio <70% and a postbronchodilator FEV1 ranging from 30% to 80% of that predicted (GOLD stages 2 and 3) [1]. Patients were enrolled into the study if they had been stable (i.e., free from exacerbations and/or requiring any changes in their treatment) for the previous 3 months. Patients with a clinical history or diagnosis of asthma were excluded from the study. During the study, patients continued to use their maintenance treatment. The study conformed to the Declaration of Helsinki, the local Institutional Ethics Committee approved the work, and informed written consent was obtained from each subject. The study was registered at clinicaltrial.gov (identifier: NCT01664767).

### 2.2. Study Design

This was a randomised, double-blinded, and controlled study that was designed to evaluate the effect of the inhalation of reducing agents (administered by inhalation of sulphurous thermal water) on airway oxidant stress and to evaluate the impact of sulphurous thermal water inhalation on clinical outcomes in COPD patients. Patients randomised to the study underwent home inhalation of sulphurous water (Acqua Breta, Terme di Riolo; Ravenna, Italy) or isotonic saline solution for 12 days. The treatment plan consisted of once daily inhalation of either sulphurous water or isotonic saline in 2 different formulations: (1) 500 mL warm inhalation, and after 20 minutes, (2) 5 mL conventional aerosolisation. This treatment plan mimics a standard onsite course of spa inhalation (warm inhalation followed by conventional aerosolisation). Sulphurous water from the springs of Riolo Terme, Ravenna (Italy) was bottled sterile in dark green glass bottles and was delivered to the Respiratory Section outpatient clinic at the University of Ferrara. Isotonic saline was bottled in similar dark green bottles. Bottle handling was performed by a person not involved in the study and who was also in charge of the randomisation procedures. Active treatment (sulphurous water) was indistinguishable from the isotonic saline (control). Twelve bottles containing sulphurous water or isotonic saline and the devices for the two inhalation procedures were delivered to the patients' homes. The investigator instructed the patients and taught them how to use the devices during the first visit. The chemical and physical characteristic of the sulphurous water and of the isotonic saline are reported in Table 1. The study consisted of three visits: recruitment (baseline), the day after the conclusion of the 12-day inhalation treatment (end of treatment), and one month after the end of the inhalation treatment (followup). At each visit, patients underwent a physical examination, lung function testing, and an assessment of their health status by the COPD Assessment Test (CAT) questionnaire. At each visit, exhaled breath condensate and sputum samples were also obtained.

### 2.3. Lung Function Test

Pulmonary function tests (Biomedin Spirometer, Padova, Italy) were performed according to published guidelines [13, 14].

### 2.4. CAT Questionnaire

The COPD Assessment Test (CAT) is an 8-item unidimensional measure of health status impairment in COPD. It was developed to be applicable worldwide and validated translations are available in a wide range of languages. In our study we used a validated Italian CAT version. The scores range from 0 to 40 [15, 16]. A CAT score >10 is found in symptomatic patients [1].

### 2.5. Sputum Processing

Spontaneous or induced sputum was collected and analysed according to standardised procedures, as previously described [17]. Sputum plugs arising from the lower respiratory tract were selected for total sputum inflammatory cell counts [18].

### 2.6. Exhaled Breath Condensate Collection and Processing

Exhaled breath condensate (EBC) was collected during oral tidal breathing using a commercial condenser (Turbo Deccs 04, Italchil, Parma, Italy) according to previous studies [19, 20]. Briefly, the subjects were not allowed to eat or drink for at least 1 h before EBC collection. Patients breathed normally through a mouthpiece for 15 min and a 2-way nonrebreathing valve that also served as a saliva trap. If the subjects felt saliva in their mouth, they were instructed to swallow it. The 50 mL Falcon collecting tubes were pre-filled with 300 *μ*L of solution consisting of 5 × 10^−2^ M cytochrome *c* from beef heart (Sigma), 5 × 10^−3^ M catalase, and Penicillin/Streptomycin (0.1 U/0.1 *μ*g), respectively, in each sample. The tubes connecting the mouthpiece to the collecting Falcon tubes were set to allow the patients to blow directly into the solution placed at the bottom of the 50 mL Falcon tubes. The harvested breath condensate was collected at –10°C, transferred to an Eppendorf tube on ice, and immediately tested for O_2_^−^ levels. The levels of O_2_^−^ were spectrophotometrically evaluated by xanthine oxidase (XO) cytochrome *c* reduction kinetics, as previously described [12, 21]. The kinetics assays were carried out in 1 mL quartz cuvettes at 37°C for 30 min in a Uvikon 860 (Kontron Instruments) spectrophotometer in the presence or absence of xanthine (0.1 mM, Sigma). Absorbance readings were taken at 550 nm (peak of reduced cytochrome). Newly generated O_2_^−^ was measured in each sample and data are expressed in micromolar concentrations. These measurements were based on the absorbance differences in the presence or absence of xanthine at the peak of the kinetic slope of cytochrome *c* reduction [12]. The data were normalised per mg of protein as determined using a Bradford assay.

### 2.7. Sample Size Calculation and Statistical Analysis

The aim of this study was to evaluate the effect of sulphurous thermal water inhalation on exhaled breath superoxide anion production. The procedures used in this study to detect superoxide anion production in exhaled breath condensate have previously been developed and internally optimised in our laboratory. A pilot analysis was conducted in stable moderate COPD patients and smokers with normal lung function to obtain reference baseline values. Based on our data, we estimated that 18 patients/group would be sufficient to detect a reduction in superoxide anion of 50%. Comparisons within groups were evaluated by ANOVA, followed, when results were significant, by using Student *t*-tests or Mann-Whitney *U* tests, as appropriate. Comparisons between groups were evaluated using a Kruskal-Wallis test, followed, when results were significant, by using Student *t*-tests or Mann-Whitney *U* tests, as appropriate. *P* values of 0.05 or less were considered to indicate statistical significance. GraphPad Prism 5.0 Software was used for the analysis.

## 3. Results

### 3.1. Study Population

Seventy-six consecutive COPD patients referred to the outpatient clinics of the Respiratory Section of the University of Ferrara were screened. Nineteen patients did not meet the inclusion criteria of the study, and seventeen patients did not choose to participate in the study. The inhalation procedures were well tolerated, and all of the patients who enrolled completed the study. No COPD exacerbations were observed during the course of the study treatments. At baseline, no difference was found in the demographic, clinical, and functional characteristics of the patients treated with isotonic saline or sulphurous thermal water (Table 2).

### 3.2. Effect of Sulphurous Thermal Water Inhalation on the Airway Oxidative Burst

**Figure 1:**
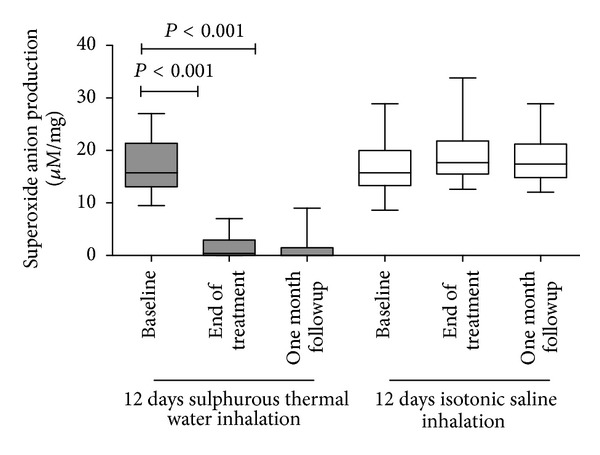
Superoxide anion production in COPD patients at the baseline, at the end of the inhalation treatment, and one month after the end of the treatment.

At baseline, a significant amount of O_2_^−^ production was observed in the exhaled breath condensate of the COPD patients after the addition of xanthine. This indicates the presence of xanthine oxidase in the exhaled breath condensate. O_2_^−^ production was virtually undetectable at the end of the 12-day inhalation treatment with sulphurous thermal water (*P* < 0.001). O_2_^−^ production was inhibited by the 12-day inhalation treatment with sulphurous thermal water irrespective of the disease severity or corticosteroid treatment (data not shown). The inhibition of O_2_^−^ production persisted for one month after the completion of the 12-day inhalation treatment with sulphurous thermal water (*P* < 0.001—Figure 1). The COPD patients who received isotonic saline inhalation showed no change in their O_2_^−^ production at the time points examined (Figure 1).

### 3.3. Effect of Sulphurous Thermal Water Inhalation on Sputum Total Inflammatory Cell Counts

**Figure 2:**
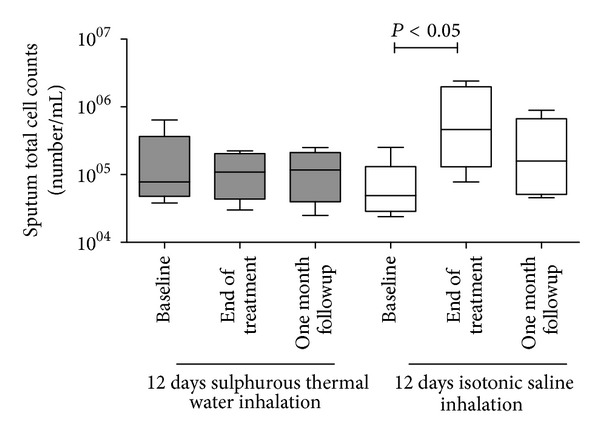
Total sputum cell counts in COPD patients at the baseline, at the end of the inhalation treatment, and one month after the end of the treatment.

At the end of the treatment and at the one-month follow-up visit, there was no difference from the baseline in the total sputum cell counts (Figure 2) in the group of COPD patients receiving sulphurous thermal water inhalation. At the end of the inhalation treatment, we observed a significant increase over the baseline in the total sputum cell counts in the group of patients receiving isotonic saline inhalation (*P* < 0.05). There was no difference in the differential inflammatory cell counts at any of the time points examined in either group (Table 3).

### 3.4. Effect of Sulphurous Thermal Water Inhalation on Lung Function

**Figure 3:**
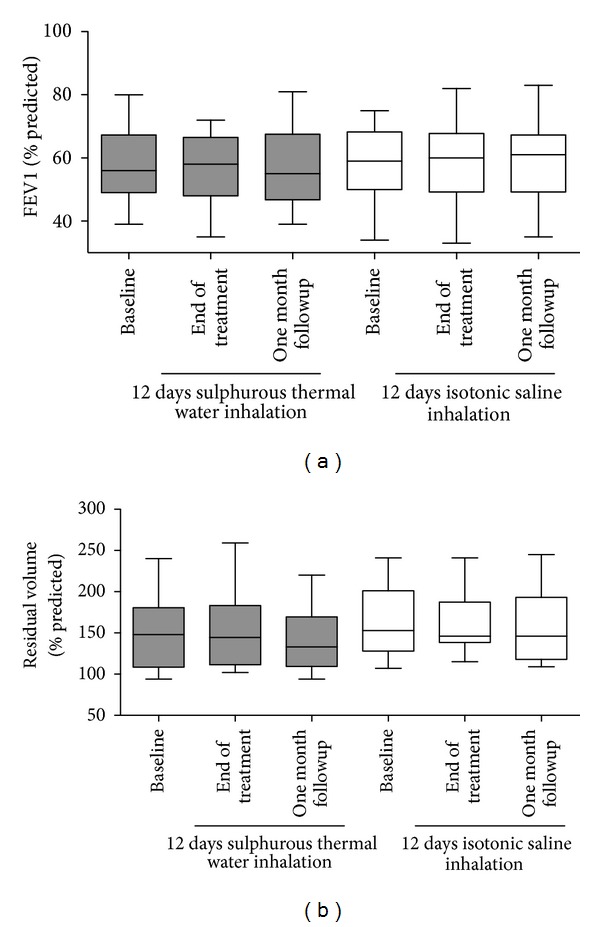
Lung function parameters (postbronchodilator FEV1 and residual volume) in COPD patients at the baseline, at the end of the inhalation treatment, and one month after the end of the treatment.

Lung function parameters did not change significantly in either group at the end of the treatment and at the one month followup when they were compared with the baseline measurements (Figure 3).

### 3.5. Effect of the Sulphurous Thermal Water Inhalation Treatment on the Symptomatic Impact of the Disease

**Figure 4:**
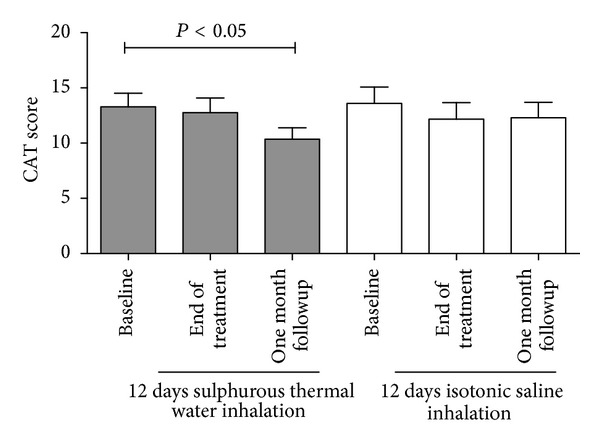
COPD Assessment Test (CAT) questionnaire in COPD patients at the baseline, at the end of the inhalation treatment, and one month after the end of the treatment.

**Figure 5:**
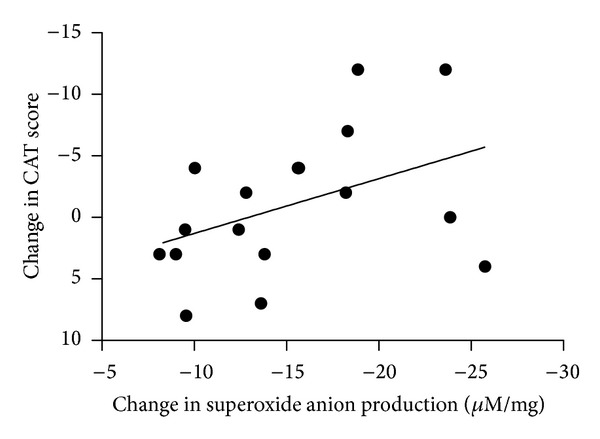
Correlation between changes in COPD Assessment Test (CAT) questionnaire score and superoxide anion production in exhaled breath condensate of COPD treated with sulphurous thermal water at one month after the end of the inhalatory treatment compared to baseline.

We observed a significant reduction in the CAT score (approximately 2.5 units) one month after the end of the sulphurous thermal water inhalation treatment compared with the score at the baseline (*P* < 0.05). We observed no differences in CAT scores in the group of patients receiving the isotonic saline as a control (Figure 4). In patients treated with sulphurous thermal water, a positive correlation was found between the improvement in CAT score and the reduction of O_2_^−^ production in the exhaled breath condensate that just missed the statistical significance (*P* = 0.08, *r* = 0.42—Figure 5). In line with the current clinical use of the thermal water inhalation in COPD patients, no major adverse events were documented.

## 4. Discussion

In this study, we found that a course of sulphurous thermal water treatment reduced oxidative stress in the airways of moderate to severe COPD patients and improved the patients' reported symptomatic impact of the disease.

Despite the wide use of thermal water inhalation treatment for chronic respiratory disorders (including COPD) the evidence supporting a clinically measurable improvement after treatment is rather weak. To date, only two placebo-controlled studies have specifically addressed this issue. Pellegrini et al. showed that a 12-day thermal water inhalation treatment resulted in a small but significant reduction in sputum neutrophilic inflammation and a short term improvement in quality of life at the end of the treatment; however, there was no short term effect on exercise capacity (measured by 6-minute walking test) or on lung function parameters [22]. Guarnieri et al. failed to document a significant reduction in neutrophil chemoattractant leukotriene B4 (LTB4) in exhaled breath condensate after a 12-day thermal water inhalation treatment but showed a significant change in exhaled breath condensate pH measurements, suggesting that thermal water inhalation can modify the biological substrate of the disease [7].

The importance of an oxidant/antioxidant imbalance in the pathogenesis of COPD is well established [2]. Thus, therapeutic strategies targeting oxidative stress with pharmacological antioxidants or designed to increase the endogenous levels of antioxidants are expected to be beneficial as an additional tool that may be used in the treatment of COPD patients. Potentially such an intervention could increase the corticosteroid sensitivity of COPD patients [23–25]. Under physiologic conditions, oxidant generation in the airways has both endogenous and exogenous sources. One of the more relevant pro-oxidative pathways of the respiratory system is the xanthine dehydrogenase/xanthine oxidase system that leads to the new generation of superoxide anion through the catabolism of the purine base xanthine [26, 27]. Increased xanthine oxidase activity has been documented in the bronchoalveolar lavage of COPD patients when compared with that of healthy subject [21, 28, 29]. It has also been shown that the treatment of COPD patients with oral allopurinol, an inhibitor of xanthine oxidase, resulted in a significant reduction in airway reactive nitrogen species [29], indicating that this intervention may be useful in reducing the inflammatory and airway oxidative burst in chronic obstructive pulmonary disease. Proteolytic and nonproteolytic pathways mediate the activation of the xanthine dehydrogenase/xanthine oxidase system [26, 27]. In particular, among nonproteolytic pathways, it has been documented that intracellular depletion of reducing agents, such as GSH, leads to xanthine dehydrogenase/xanthine oxidase system activation [30, 31]. We have previously reported that *in vitro* stimulation of bronchial epithelial cells with sulphurous thermal water results in increased intracellular GSH levels [12]. In this study, we found a significant *in vivo* reduction of xanthine-induced superoxide anion production following a course of reducing agents that were delivered by sulphurous thermal water inhalation. Taken together these data suggest that the inhalation of sulphurous thermal water results in increased intracellular antioxidant levels that are able to modulate or inhibit the activation of the xanthine dehydrogenase/xanthine oxidase system, thereby decreasing the airway oxidative burst in COPD patients. Thus, modulation of xanthine oxidase may represent a therapeutic option for decreasing oxidative stress in the lungs of COPD patients. Consistent with a previous study [22], we observed an increased number of total sputum cells after the 12-day course of saline inhalation treatment as a control. The increased recovery of cells in the induced sputum that we observed after the treatment is in line with the observations made in COPD patients whose inhalation of isotonic or hypertonic solutions administered by nebulisation induces increases in airway secretions that can be expectorated [32]. The increased recovery did not occur after 12-day sulphurous water inhalation, suggesting that this inhalation does not affect airway secretion and/or inflammation. Interestingly we also documented that (i) the antioxidant effect persisted for up to one month after the end of the inhalation treatment period; and (ii) the antioxidant effect was paralleled by a significant improvement in the patient's reported symptomatic impact of COPD, supporting an interplay between the modulation of the oxidative burst and an improvement in clinical outcomes in these patients.

In line with previous findings [22], we failed to document any improvement in lung function following the thermal water inhalation treatment in COPD patients. Pellegrini et al. previously reported a short term improvement in the quality of life of COPD patients following the thermal water inhalation treatment [22]. We now extend these findings by reporting a significant improvement in symptoms that persist for one month after the end of the inhalation treatment.

Although we cannot exclude that some patients might have perceived the thermal vapour characteristics, it is unlikely that this affected the result of the study since the improvement in the symptomatic impact of the disease was not observed immediately after the end of the inhalation treatment but it appeared one month after its completion. This reinforced the reliability of the conclusion. However, the link between thermal water inhalation treatment and an improvement in the patient's reported quality of life in COPD patients remains largely unexplored.

In conclusion, thermal water inhalation treatment is a safe procedure leading to *in vivo* antioxidant effects in COPD patients. Thermal water inhalation treatment in COPD patients results in positive clinical effects in self-reported quality of life that persists one month after the end of the treatment. Larger studies are needed to confirm the positive clinical findings of thermal water inhalation treatment in COPD patients. Even more importantly, studies with a longer follow-up time frame up are required to evaluate whether inhalation of thermal water is able to modify relevant clinical outcomes of the disease.

## Figures and Tables

**Table 1:** Physical and chemical characteristics of sulphurous water and isotonic saline.

Parameters	Sulphurous water	Isotonic saline
pH	7.1	4.5–7.0
Ca_2_^+^ g/L	0.251	Absent
Mg_2_^+^ g/L	0.091	Absent
NH_4_^+^ g/L	0.00075	Absent
HCO_3_^−^ g/L	0.523	Absent
SO_4_^−^ g/L	0.5	Absent
H_2_S g/L	3.83	Absent

**Table 2:** Characteristics of the study population at the baseline.

	Study population	Patients treated with inhaled sulphurous water	Patients treated with inhaled isotonic saline	*P* value*
*n*	40	20	20	
Age	69.9 ± 1.0	69.3 ± 1.5	70.4 ± 1.4	ns
Sex (male/female)	29/11	14/6	15/5	ns
Smoking habit				
Pack/years	39.7 ± 2.2	38.5 ± 3.2	41.1 ± 2.2	ns
Current smokers (*n*)		1	1	
Exsmokers (*n*)		19	19	
Nonsmokers (*n*)		0	0	
Prebronchodilator FEV1 (litre)	1.38 ± 0.06	1.41 ± 0.09	1.35 ± 0.08	ns
Prebronchodilator FEV1 (%)	54.3 ± 1.8	54.9 ± 2.6	53.6 ± 2.5	ns
Postbronchodilator FEV1 (litre)	1.48 ± 0.07	1.52 ± 0.10	1.44 ± 0.08	ns
Postbronchodilator FEV1 (%)	58.1 ± 1.8	58.9 ± 2.7	57.4 ± 2.6	ns
Reversibility to bronchodilator (litre)	0.10 ± 0.01	0.11 ± 0.01	0.09 ± 0.01	ns
Reversibility to bronchodilator (%)	6.7 ± 0.4	6.8 ± 0.5	6.6 ± 0.7	ns
Residual volume (%)	154.4 ± 7.4	149.5 ± 10.7	156.5 ± 10.3	ns
Use of long acting antimuscarin (%)	11	5	6	ns
Use of long acting *β*2-agonist (%)	38	18	18	ns
Beclomethasone (BDP) equivalent daily dose (*μ*g)	1545 ± 110	1571 ± 159	1520 ± 157	ns
Use of inhaled corticosteroids (*n*)	29	15	14	ns
CAT score	13.4 ± 0.9	13.3 ± 1.2	13.6 ± 1.4	ns

*Patients treated with inhaled sulphurous water versus patients treated with inhaled isotonic saline.

**Table 3:** Inflammatory cell counts in the sputum of COPD patients.

	Sulphurous water inhalation			Isotonic saline inhalation		
	Baseline	End of treatment	One month followup	Baseline	End of treatment	One month followup
Macrophages (%)	25 ± 11	26 ± 16	19.3 ± 13	19.5 ± 8.6	17.9 ± 12.8	20.1 ± 9.8
Neutrophils (%)	72.8 ± 10.0	74 ± 16	78.9 ± 12	79.9 ± 4.6	82.1 ± 14.3	78.8 ± 11.8
Eosinophils (%)	1.2 ± 0.6	0	1.1 ± 0.4	0.6 ± 0.3	0	0.5 ± 0.2
Lymphocytes (%)	0.3 ± 0.1	0	0.7 ± 0.3	0	0	0.6 ± 0.3
